# Priority Areas for Child Diaper Access: Low-Income Neighborhoods with Limited Retail Access to the Basic Need of Diapers

**DOI:** 10.1089/heq.2021.0192

**Published:** 2022-09-27

**Authors:** Kelley E.C. Massengale, Melissa A. Jones, Juncheng Liao, Christine Park, Michelle Old

**Affiliations:** ^1^ Diaper Bank of North Carolina, Durham, North Carolina, USA.; ^2^ Department of Informatics and Analytics, University of North Carolina at Greensboro, Greensboro, North Carolina, USA.; ^3^ Department of Statistical Science, Duke University, Durham, North Carolina, USA.; ^4^ Duke University School of Medicine, Durham, North Carolina, USA.; ^5^ Diaper Bank of North Carolina, Durham, North Carolina, USA.

**Keywords:** diaper need, hygiene product access, diaper banks, infant health, child health, material hardship

## Abstract

**Introduction:**

Although a requirement for the health and hygiene of young children, millions of US families with low-incomes have unmet needs for diapers. The present study explored retail options in Durham County, NC for purchasing diapers in low-income neighborhoods in effort to increase our understanding of the overall context of diaper need.

**Methods:**

During June 2018, we visited 63 retailers selling 2460 child diaper products in 29 census tracts with a median household income ≤200% of the federal poverty guideline. Corner stores were the only retailers to sell products without original packaging, including one corner store selling loose diapers for $1.49 each. Next, we calculated bus routes to determine accessibility of the retailer with the lowest prices and greatest selection. One-way bus travel from all other census tracts to a big-box store required taking two buses combined with an average of 11 min walking for an average travel time of 43 min. We deemed census tracts as “priority areas for diaper access” when they were characterized as: (1) low income and (2) low access with no retailer selling all of the 10 most common child diaper sizes.

**Results:**

Nearly half (*n*=13) of the census tracts in our sample met our criteria for priority areas. We compared neighborhood characteristics of priority areas with all other county census tracts. Families living in priority areas were statistically significantly more likely to: identify as Black or African American, face challenges affording housing costs, have homes or automobiles in need of repair, experience neighborhood violence, and have less educational attainment.

## Introduction

Although a requirement for the health and hygiene of infants and toddlers, millions of families in the United States with low incomes have unmet needs for diapers.^1^ Diaper need forces families to make decisions about whether to spend money on diapers or on another basic needs, to have a supply of diapers adequate for changes at healthy intervals.^1–6^ An issue of public health concern, the experience of diaper need negatively impacts physical health for children (e.g., susceptibility to skin and urinary tract infections as well as poor sleep) and mental health for parents and caregivers.^1–10^ In an effort to save money on diapers, parents and caregivers may attempt to toilet train early, before children show signs of developmental readiness.^3,5^

Although state and federal policy programs address some needs for food, housing, and health care, no such program exists to help families meet the basic need of diapers. Families experiencing diaper need have reported coexisting forms of material hardship, including food insecurity and challenges meeting needs for: housing, transportation, other hygiene products, education, utilities, and medical bills.^2–4^

Families without an adequate diaper supply are excluded from full participation in society, impacting their abilities to leave home to attend community events and influencing attendance in both workplace and educational settings. During the fourth trimester (i.e., the postpartum period), women with low incomes are less likely to have access to paid maternity leave compared with women with higher paying jobs, and therefore less income to allocate toward basic needs such as diapers.^11^ Childcare providers generally require families to provide disposable diapers for the time children are in their care.^3,5,9^

When families cannot meet childcare providers' diaper requirements, families may be forced to keep their children at home instead.^3,5,9^ The lack of childcare may then force parents to miss work, further limiting household income.^3,5,9,^ A Connecticut study found that parents who could not meet childcare providers' diaper requirements missed an average of 4 days of work or school each month, attributable to diaper need.^12^ Parents' long-term income earning potential may be impacted by experiences of diaper need when their children are young.^12^

The average cost of a month's diaper supply, $100, presents a significant burden to families in poverty.^5^ To meet this demand, the poorest 20% of U.S. families spend 14% of their income on diapers compared with only 1% for the richest 20% of families.^13^ Families with the least income often pay higher prices when shopping for food or household necessities.^14,15^ The “poverty penalty” occurs when families with low incomes pay more for something than others with higher incomes.^16^ Money saving strategies may be inaccessible for families with low incomes.

For example, bulk shopping clubs require membership fees; online purchases require a credit card, Internet, and an address for secure deliveries; comparison shopping may necessitate transportation; and purchasing large quantities requires more money upfront.^5^ Families who can spend more at once may receive discounts for purchasing: multiple items, items in larger quantity, or products “on sale” even if they are not immediately needed.^15,16^

With nearly half of U.S. families living within 200% of the federal poverty guideline, an accessible and affordable option for meeting diaper need is essential.^1^ Diaper banks, nonprofit organizations working to provide a supplemental supply of diapers to families in need, do not provide all the diapers required nor do they exist in all communities.^1,3^ Recipients must purchase or acquire additional diapers to change diapers at healthy intervals. Research on the food shopping habits of families in census tracts with low incomes and low access to food found that families with the lowest incomes who received food from community food sources (e.g., food pantries and social service organizations), shopped at grocery stores least often, and utilized public transportation or borrowed a vehicle to shop were those most likely to report problems with food access.^17^

The researchers concluded that understanding retail food access and utilization of community food resources was important for informing policies addressing food insecurity in low-income census tracts.^17^ A similar understanding about retail access to diapers in low-income census tracts in a county with a community-based diaper bank may serve to inform policies to address diaper need. There is a gap in the literature to document retail availability of diapers in low-income neighborhoods where families experiencing diaper need may live.

Therefore, the purpose of the present study was to explore the landscape of retail options available to Durham, NC families purchasing diapers in low-income census tracts. Our goal was to better understand what options local families with low incomes may have for purchasing diapers near their homes in an effort to increase our understanding of the overall context of diaper need. The findings from this study have implications for advancing access to health services, neighborhood planning, policies to address diaper need, and health equity.

## Methods

With the goal of documenting what retail options families with low incomes would have for purchasing diapers in the census tracts where they lived, we developed a comprehensive listing of all retail locations selling diapers in census tract with a median household income ≤200% of the poverty guideline. Next, we identified which census tracts provided low access to retail diapers and then compared the characteristics of those census tracts with others providing more retail options.

### Community of interest

Per the U.S. Census, Durham County, NC is home to 316,739 people with a median household income of $56,393.^18^ Residents identified as: White (55%), Black or African American (37%), Asian (6%), or another racial identity (4%). Fourteen percent of residents also identified as Hispanic or Latino. Nearly half of (46.1%) families with children lived in households whose income was ≤200% of the federal poverty guideline.^19^ A community survey of Durham County residents found that 88% use automobile transportation for the local trip they take most often, whereas only 9% take the bus.^20^ However, regular and occasional bus passengers were more likely to have low incomes than residents who did not ride the bus.^20^

### Geographic sample

We identified all Durham County, NC census tracts in which the median household income was ≤$50,200, 200% of the U.S. Department of Health and Human Services 2018 poverty guideline for a family of four.^21^ Of the 59 census tracts, 29 were ≤200% of the poverty guideline (i.e., low income) and therefore included in our sample. Utilizing windshield survey and walking survey techniques, we developed a landscape listing of diaper retailers in each census tract by driving or walking to every retail location in each census tract that potentially sold diapers and documenting what diapers products (if any) were sold.^22^

To maximize efficiency and accuracy during our time driving, we first toured each census tract virtually.^23^ Using Google Street View maps, we explored each census tract, listing addresses for potential diaper retailers.^24^ In June 2018, we visited each retailer in person, stopping at additional retailers identified en route. Retailers identified during virtual or driving tours who did not sell child diapers upon our physical visit to the store were not included among the list of diaper retailers.

### Retail visits

At each retailer, all diaper products were photographed or video recorded. For each item sold, we recorded the product brand and description, product size, package quantity, and package price. If an item was temporarily out of stock (e.g., price tag was present but the item was absent), we included the item among those sold. In total, 63 retailers sold 2460 child diaper products.

#### Retailers

Retailers selling diapers were categorized as: big-box store (e.g., physically large retail store offering a range of products without requiring a paid membership to shop), corner store (e.g., mini-mart or tienda), discount retailer (e.g., dollar store), drug store, or grocery store. We excluded a private membership warehouse club that required a paid membership to shop.

#### Brands

Diaper brands were categorized as: name brand (e.g., Huggies, Pampers, Luvs, GoodNights), store brand, natural brand (e.g., Babyganics, Honest, Seventh Generation, Simple Truth), or generic. Although store brand diapers tend to be cheaper than name brand diapers, which tend to be cheaper than natural brand diapers, parents and caregivers sometimes develop a preference for particular brands that they may feel are of better quality, fit, or leak less often.

#### Size, quantity, and cost

We noted whether each retailer sold the 10 most common child diaper sizes (i.e., newborn, size 1, size 2, size 3, size 4, size 5, size 6, 2T/3T pull-ups, 3T/4T pull-ups, and 4T/5T pull-ups). We also included in the “total number of diaper products sold per retailer” products smaller or larger than these sizes (e.g., “preemie” size for low birthweight infants or overnight products for older children). In the face of diaper need, families may use a diaper larger than the size recommended for their child's weight if they do not have a diaper supply sufficient to change their child's diaper as often as recommended.^3,8^ As diaper bank recipients request size 5 diapers most often, we calculated each retailer's lowest cost per size 5 diaper.

#### Priority areas

The U.S. Department of Agriculture identifies census tracts as food deserts when they are both low income and provide low access to healthy food.^25^ Similarly, we deemed census tracts as priority areas for diaper access when they were characterized as:

(1) *Low income*: the average median household income for the census tract was within 200% of the federal poverty guideline for a family of four *and*
(2) *Low access*: no retailer in the census tract sold all of the most common child diaper sizes.

#### Bus route

We identified a big-box store as the retailer in our sample that provided the: (1) lowest cost per individual diaper, (2) cheapest cost per individual size 5 diaper, and (3) largest number of diaper products sold. Therefore, this store represents the retail location that a family could visit with the greatest likelihood of finding diapers in the size(s) needed and at the lowest price. To determine accessibility of the big-box store via public transportation, we calculated the bus route for a one-way trip from every other retailer to this location. For consistency, each route was calculated using the “Plan a Trip” feature on the website of GoDurham, the local public transit system, for a trip departing within an hour from 5 pm on a non-holiday Monday.^26^

When multiple routes were available, preference was given to the option with the shortest total travel time as we surmised that families would opt for the quickest route. For each census tract, we recorded the number of buses and number of minutes walking required to complete a one-way trip to the big-box store from the retailer with the shortest travel time. In census tracts in which no retailers sold diapers, one-way trips were calculated from all other retailers visited, recording the route with the shortest total travel time as this route may represent the selection of someone seeking to spend the least amount of time traveling.

### Neighborhood characteristics

Characteristics of each census tract were identified using the publicly available, open-source platform, Durham Neighborhood Compass, a tool created by nonprofit DataWorksNC.^27^ The Durham Neighborhood Compass compiles data from local health systems, the census, state and federal agencies, and local government. Using two-sample *t*-tests, we compared neighborhood characteristics of the census tracts identified as priority areas for diaper access to all other census tracts in the county.

Comparisons were made between the two groups on resident-reported racial and ethnic identities, percentage of college degrees earned, average age of death, infrastructure access (e.g., homes near bus stops and rates of sidewalk length to roadway length), maintenance of housing and automobiles (e.g., percentage of residential properties in poor or unsound state of repair, number of housing code violations, unmaintained property violations, automotive code violations), ownership of residential properties, and crime rates.

## Results

### Retail options

The number of retailers in each census tract selling diaper products ranged from 0 to 7 (Table 1). Among retailers, the number of products for sale ranged from a single option to nearly 200 (generated from size and brand options). Retailers varied in the number of brands on offer.

**Table 1.** Summary of Infant and Toddler Diaper Retail Availability, by Durham, North Carolina Census Tract

Census tract	Retailer types	No. of Retailers^a^	No of products sold, range^b^	Common sizes available	Brand types available	Range package size^c^	Min. cost^d^	Max. cost^d^	Cheapest cost, size 5^d^	Quickest one-way route to big-box store
1.1	Discount retailer, grocer	2	6–54	Yes	Name, generic	12–132	$0.13	$0.78	$0.18	2 buses +9 min. walking=47 min
1.2	Drug store	2	49–88	Yes	Name, natural, store	11–108	$0.14	$1.27	$0.24	2 buses +8 min walking=53 min
2	Grocer	1	8	No	Generic	14–36	$0.19	$0.50	$0.26	2 buses +9 min walking=50 min
3.1	None available	0	0	No	None available	0	N/A	N/A	N/A	2 buses +10 min walking=47 min
4.2	Corner store, discount retailer, grocer	7	2–130	Yes	Name, natural, store, generic	11–198	$0.10	$1.02	$0.18	2 buses +8 min walking=32 min
5	Corner store, grocer	3	2–18	No	Name, natural, generic	18–64	$0.21	$0.59	$0.40	1 bus +12 min walking=29 min
6	Corner store, discount retailer, grocer	3	1–81	Yes	Name, store, generic	11–132	$0.11	$1.00	$0.18	1 bus +9 min walking=16 min
7	Grocer	1	25	Yes	Name, generic	14–56	$0.17	$0.73	$0.21	1 bus +15 min walking=34 min
9	None available	0	0	No	None available	0	N/A	N/A	N/A	2 buses +9 min walking=40 min
10.1	Corner store	1	15	No	Name, generic	14–48	$0.25	$0.83	$0.43	2 buses +8 min walking=44 min
10.2	Discount retailer, drug store, grocer	4	3–49	Yes	Name, store, generic	4–132	$0.13	$1.18	$0.20	2 buses +9 min walking=48 min
11	Grocer	2	9–31	No	Name, generic	14–104	$0.12	$0.67	$0.21	2 buses +10 min walking=41 min
13.1	None available	0	0	No	None available	0	N/A	N/A	N/A	2 buses +5 min walking=43 min
13.3	None available	0	0	No	None available	0	N/A	N/A	N/A	2 buses +9 min walking=51 min
13.4	Corner store, grocer	3	2–72	Yes	Name, store, generic	11–132	$0.16	$1.00	$0.18	1 bus +29 min walking=36 min
14	Corner store, discount retailer	3+	1–50	Yes	Name, generic	1–132	$0.16	$1.49	$0.18	2 buses +9 min walking=49 min
15.1	None available	0	0	No	None available	0	N/A	N/A	N/A	1 buses +14 min walking=26 min
15.2	None available	0+	0	No	None available	0	N/A	N/A	N/A	1 bus +9 min walking=21 min
17.1	Discount retailer	1+	3	No	Generic	4–5	$0.20	$0.25	N/A	2 buses +10 min walking=58 min
17.11	Discount retailer, drug store, grocer	6	3–82	Yes	Name, natural, store, generic	4–148	$0.11	$1.18	$0.18	2 buses +9 min walking=58 min
17.6	None available	0+	0	No	None available	0	N/A	N/A	N/A	2 buses +7 min walking=41 min
17.9	Discount retailer, grocer	2	50–72	Yes	Name, store, generic	11–132	$0.13	$1.00	$0.18	2 buses +11 min walking=54 min
18.2	Corner store, discount retailer, grocer	4+	1–72	Yes	Name, store, generic	10–132	$0.13	$1.00	$0.18	2 buses +9 min walking=47 min
20.15	Big-box store^*^	1	173	Yes	Name, natural, store	11–176	$0.12	$0.82	$0.17	—
20.16	Corner store, discount retailer; drug store; grocer	6	2–88	Yes	Name, natural, store, generic	4–148	$0.16	$1.27	$0.22	8 min walking=8 min
20.26	Corner store, discount retailer	2+	1–3	No	Generic	4–24	$0.20	$0.25	N/A	2 buses +5 min walking=53 min
20.27	Drug store, grocer	2+	49–72	Yes	Name, natural, store	11–132	$0.14	$1.18	$0.18	2 buses +16 min walking=54 min
20.9	Discount retailer, drug store	2+	50–88	Yes	Name, natural, store, generic	11–132	$0.13	$1.27	$0.18	2 buses +10 min walking=60 min
23	Corner store, discount retailer, drug store, grocer	4	2–50	Yes	Name, store, generic	10–132	$0.13	$1.18	$0.18	1 bus +16 min walking=36 min

^a^ Identified as a food desert per the U.S. Department of Agriculture.^25,27^

^b^ Per retailer.

^c^ Range number of diapers per package sold.

^d^ Cost per individual diaper^*^Retailer in our sample that provided the lowest cost per individual diaper product, the cheapest cost per individual size 5 diaper, and the largest number of diaper products for sale.

#### Big-box store

**FIG. 1.**
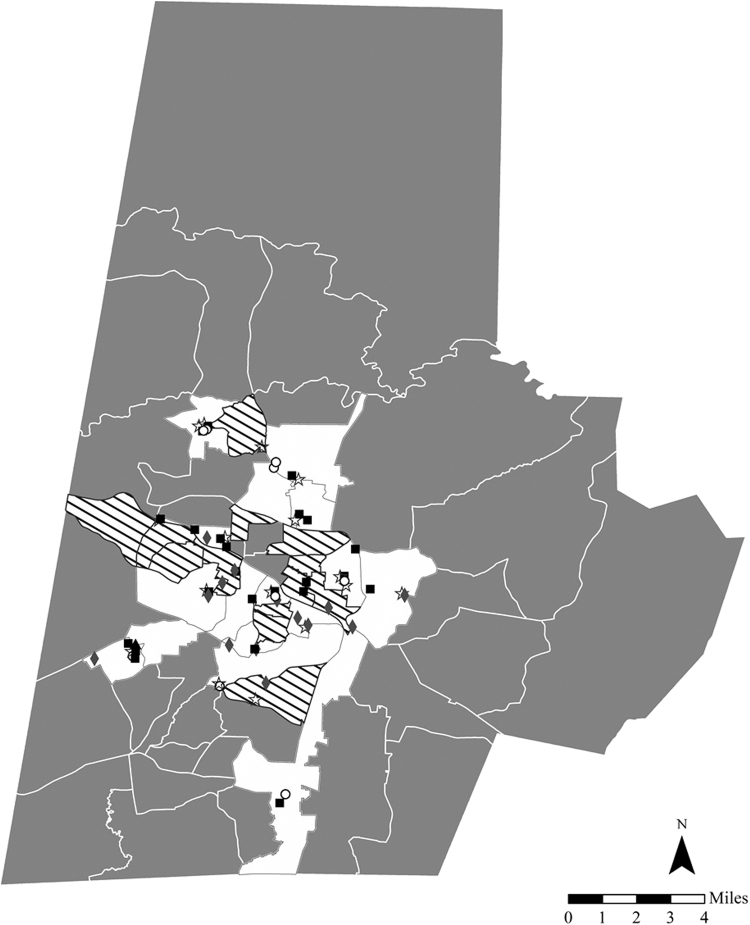
Durham County, NC low-income census tracts and diaper retail locations. 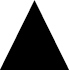 Big box store; 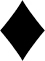 Corner store; 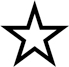 Discount retailer; 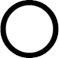 Drug store; 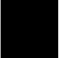 Grocer; 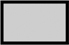 Median household income ≤200% federal poverty guideline. 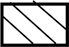 Priority area for diaper access.

The one big-box store in our sample provided the largest selection of products, the cheapest cost per size 5 diaper, and the lowest cost for bulk diaper purchases (Fig. 1). Name brand, natural brand, and store brand products were sold.

#### Corner stores

The 14 corner stores in our sample each sold 1 to 18 diaper products. Most corner stores sold only generic products, whereas a few sold brand name products only or brand name and generic products. No natural brand or store brand products were available. Compared with other types of retailers, corner stores were less likely to sell cases of diapers, selling instead smaller quantities. Most sold only a few diaper sizes, whereas the largest number of sizes sold was 8. Only half of corner stores sold size 5 diapers; the average cost of the cheapest size 5 diaper available was $0.50.

Corner stores were the only retailers in our sample to sell products that had been removed from their original packaging. One corner store sold individual diapers without any packaging for $1.49 each. Two corner stores sold individual sleeves of diapers that had been removed from factory sealed boxes containing multiple sleeves.

#### Discount retailers

The 16 discount retailers in our sample each sold 3 to 75 diaper products. A quarter sold name brand and store products; nearly half sold name brand and generic products; and the rest sold generic products only. No natural brand products were available. Among the discount retailers, small packages containing as few as 4 diapers were sold as well as cases containing 132 diapers. Two-thirds of the discount retailers sold all of the most common diaper sizes, with 11 retailers selling size 5 diapers. The average cost of the cheapest size 5 diaper sold by a discount retailer was $0.19 per diaper.

#### Drug stores

The nine drug stores in our sample each belonged to one of two different national chains. One chain sold 49 diaper products, and the other sold 88. All sold name brand and store brand products, with a third also selling natural brand diapers. The quantity of diapers per package ranged from 11 to 108. Each sold the most common sizes. The average cost of the cheapest size 5 diaper from each retailer was $0.25.

#### Grocers

The 23 grocery stores in our sample sold between 5 and 130 diaper products each. Stores sold a combination of brands, with a few selling only name brand, only natural brand, or generic only. One-third sold a mix of name brand and generic, whereas another third sold name brand and store brand diapers. Only one grocer sold name brand, natural brand, and store brand products. The quantity of diapers per package ranged from 11 to 198. Sixty percent sold all of the most common sizes. All but two grocers sold size 5 diapers. The average cost of the cheapest size 5 diaper available per grocer was $0.26.

#### Priority areas for diaper access

Nearly half of the census tracts in our sample met our criteria for priority areas for diaper access. All had an average median household income within 200% of the federal poverty guideline for a family of four and low access to diapers given that none of the retailers sold all the most common sizes. In half of the priority areas, no retailers sold any diaper products (i.e., the corner stores, gas stations, grocers, and discount retailers selling food products in these census tracts did not sell diapers). In only four of the priority areas were size 5 diapers sold, ranging in price ($0.21–$0.43 per diaper). The retailers in the priority areas included corner stores, discount retailers, and grocers.

#### Bus routes

We assessed the accessibility of the big-box store via public transportation. From one neighboring census tract, a person could potentially walk to the store. Travel to the big-box store *one-way* by bus from all other census tracts required in most cases taking two buses combined with an average of 11 min walking for an average travel time of 43 min. Fare for a one-way bus trip cost $1.00 (regular fare) or $0.50 (discounted fare).

### Neighborhood characteristics of priority areas

Compared with other census tracts, residents of priority areas for diaper access were more likely to identify as Black or African American (50.91% vs. 34.02%, *p*=0.01), less likely to identify as White (25.18% vs. 46.73%, *p*<0.01), and less likely to have earned a Bachelor's degree (26.49% vs. 44.01%, *p*=0.01) (Table 2). Priority areas were better served with bus stops (93.00% vs. 56.80%, *p*<0.001) and more likely to have sidewalks alongside roads (51.65% vs. 35.28%, *p*<0.01). Residential properties in priority areas were more likely to be occupied by a renter (72.00% vs. 44.39%, *p*<0.001) and less likely to be maintained to housing code (83.04 violations per square mile vs. 13.15, *p*=0.046).

**Table 2.** Neighborhood Characteristics of Priority Areas for Diaper Access

Neighborhood characteristic	Priority areas	Non-priority areas	***p*** ^a^
Demographics			
Asian	7.00%	4.87%	0.25
Black or African American	50.91%	34.02%	**0.01**
Hispanic or Latino	15.80%	12.69%	0.33
White	25.18%	46.73%	**0.004**
Adults with a Bachelor's degree or more	26.49%	44.01%	**0.01**
Average age of death (years)	67.40	70.05	0.10
Infrastructure			
Homes near bus stops	93.0%	56.8%	**<0.001**
Rate of sidewalk length to roadway length	51.65%	35.28%	**0.01**
Housing and personal property			
Residential properties in poor or unsound state of repair	2.07%	0.50%	**0.048**
Violations in minimum housing code, per square mile	83.04	13.15	**0.046**
Unmaintained property violations, per square mile	191.41	26.93	**0.03**
Renter-occupied housing	72.00%	44.39%	**<0.001**
Cost-burdened renters	53.66%	44.29%	**0.01**
Cost-burdened mortgage holders	44.27%	25.24%	**0.03**
Automotive code violations, per square miles	27.71	7.28	**0.03**
Neighborhood violence (per square mile)			
Property crimes	294.78	148.97	**0.009**
Drug crimes	33.98	10.78	**0.02**
Violent crimes	119.05	41.25	**<0.001**

Bold value indicates statistically significant.

^a^ *p* Values obtained from two-sample *t*-tests. Assumptions of normality were assessed.

More renters (53.66% vs. 44.29%, *p*<0.01) and mortgage holders (44.27% vs. 25.24%, *p*=0.03) were cost-burdened, (i.e., spending >30% of their annual household income on rent or mortgage payments), than renters and mortgage holders in other parts of the county. Automobile code violations were present at more than three times the rate of violations in census tracts that were not identified as priority areas for diaper access (*p*=0.03). All categorized crimes occurred at higher rates per square mile in priority areas (property crimes 294.78 vs. 148.97, *p*<0.01; drug crimes 33.98 vs. 10.78, *p*=0.02; violent crimes 119.05 vs. 41.25, *p*<0.001). The average age of death was 3 years younger in priority areas, although this was not statistically significant.

## Discussion

Census tracts we identified, as priority areas for diaper access were statistically significantly more likely than other census tracts to contain: cost-burdened renters or mortgage holders, homes or automobiles in need of repair, and reports of neighborhood violence. Priority area census tract residents were also more likely to be Black or African American, less likely to be college graduates, and less likely to own their homes, a likely consequence of historical redlining in the community.^28^ Documenting the retail landscape of diaper access in the context of neighborhood inequities shows how retail access to diapers may present an additional challenge to families living in neighborhoods that may be impacted by the current and historical systemic disparate distribution of wealth, community resources, and educational opportunities.

Families likely face competing priorities for their income, including rent or mortgage payments, automobile and/or home repairs, and meeting other basic needs. Renters may have landlords who have failed to adequately maintain the dwellings they rent or make needed repairs. The sociodemographic characteristics of priority areas are similar to documented characteristics of food deserts.^29,30^ Understanding local retail access to diapers among families with low incomes is an important step in achieving equity and health justice for young children.

Families in low-income communities depend upon discount retailers, corner stores, and drug stores (i.e., nontraditional food stores) as places to purchase food, often citing proximity as the impetus.^14^ We expect that families would also purchase diapers at these same locations. Corner stores and discount retailers, which made up nearly half of the retailers in our sample, were more likely to sell diapers in smaller quantities (e.g., “loose” diapers or small packages containing four diapers) than other types of retailers. In the way that single “loosie” cigarettes are illegally sold in low-income communities,^31^ so too are infant diapers.

Given the diapering needs of young children, families would need multiple small packages of diapers to meet even a day's need. Meeting the entirety of a child's diaper needs from the offerings of a corner store or discount retailer is akin to using only travel-sized toothpaste for oral health. Corner stores also offered, on average, higher prices per diaper than other retailers, which was not surprising as these are also locations where families typically pay higher prices for food items and for packages of smaller quantities of other essentials.^14,15^ To pay the cheapest diaper prices would require most families in our community of interest to travel outside their census tract.

In a community in which most people travel by personal vehicle and most bus riders have low incomes,^20^ families without access to a private vehicle may be those who are most negatively impacted by a lack of retail access to diapers in their neighborhoods. Although the neighborhoods in priority areas for diaper access were better served by bus than non-priority areas, we surmised bus travel to a big-box store for diaper purchasing presents challenges just as families living in food deserts cited transportation as a barrier to visiting grocery stores outside their neighborhoods.^32^

The logistics of a bus journey with small children, especially when a bus transfer and significant walk are also required, is compounded by the necessity of carrying back a bulky diaper case. Bus fare to the big-box store in our sample with both the largest range of diaper products for sale and the cheapest prices costs the equivalence of half a day's worth of diapers.

Our findings on the time commitment required to access the big box store by bus document that for families with potentially the lowest incomes and greatest challenges to meeting their basic needs, both the convenience of having nearly 200 diaper products for sale at one location and the option to buy diapers at the cheapest prices may be inaccessible. These findings offer another example of how families with more income may pay less for diapers and spend less time meeting their basic needs.

### Limitations

Our study documents the retail availability of child diapers in low-income neighborhoods but did not ask families where or how (e.g., in-person or online) they shop. Future research is needed that includes the voices of families with low incomes to better understand their purchasing experiences and to investigate whether increasing local access to affordable diapers improves health outcomes.

### Health equity implications

Diapers are an essential need for young children, the cost of which may be prohibitive for families with low incomes.^1^ Families without access to diapers are unable to participate in the community outside of home, including attending childcare, community events, and work. Priority areas for diaper access exist in low-income neighborhoods where residents do not have proximal access to retailers selling the most common child diaper sizes. Families purchasing diapers in low-income census tracts are likely to pay higher prices per individual diaper than families able to access big-box stores for cheaper products.

Higher prices paid to meet the basic need of diapers leave less income for other basic needs and health-related expenses. To address this public health concern, families need access to a diaper supply adequate for allowing diaper changes at intervals required for health maintenance.^1–8^ Families whose diaper need is addressed experience a range of benefits that impact their health, household economics, participation in social service programs, and parents' confidence in their own parenting.^3,33–35^ Facilitating such diaper access requires opportunities to purchase diapers at affordable prices at local retailers; expanded access to the safety net provided by nonprofit diaper banks; and local, state, and federal policies to address unmet hygiene needs.
